# Optimising assessment of dark adaptation data using time to event analysis

**DOI:** 10.1038/s41598-021-86193-3

**Published:** 2021-04-15

**Authors:** Bethany E. Higgins, Giovanni Montesano, Alison M. Binns, David P. Crabb

**Affiliations:** 1 Optometry and Visual Sciences, School of Health Sciences, City, University of London Northampton Square, London, EC1V 0HB UK; 2 Moorfield’s Eye Hospital NIHR Biomedical Research Centre, Moorfields Eye Hospital NHS Foundation Trust and UCL Institute of Ophthalmology, London, UK

**Keywords:** Health care, Medical research

## Abstract

In age-related macular degeneration (AMD) research, dark adaptation has been found to be a promising functional measurement. In more severe cases of AMD, dark adaptation cannot always be recorded within a maximum allowed time for the test (~ 20–30 min). These data are recorded either as censored data-points (data capped at the maximum test time) or as an estimated recovery time based on the trend observed from the data recorded within the maximum recording time. Therefore, dark adaptation data can have unusual attributes that may not be handled by standard statistical techniques. Here we show time-to-event analysis is a more powerful method for analysis of rod-intercept time data in measuring dark adaptation. For example, at 80% power (at α = 0.05) sample sizes were estimated to be 20 and 61 with uncapped (uncensored) and capped (censored) data using a standard t-test; these values improved to 12 and 38 when using the proposed time-to-event analysis. Our method can accommodate both skewed data and censored data points and offers the advantage of significantly reducing sample sizes when planning studies where this functional test is an outcome measure. The latter is important because designing trials and studies more efficiently equates to newer treatments likely being examined more efficiently.

## Introduction

Dark adaptation (DA) is recovery of light sensitivity in a dark environment after exposure to bright light which has bleached a significant proportion of visual pigment^1^. There is accumulating evidence that the impairment of DA is a functional measure of age related macular degeneration (AMD)^1–10^. This impairment has been found to increase with presence of reticular pseudodrusen^11^. The time taken for DA to occur is dependent upon the rate at which visual pigment is regenerated by the outer retina, a process in which the retinal pigment epithelium plays a central role^12^. Abnormal DA results in a slowed recovery of photoreceptor sensitivity following exposure to a bleaching light source. Measurable aspects of DA include the rates of rod and cone recovery and the time taken to reach the rod cone break^13^.

Measuring DA is fraught with issues around excessive test duration and minimal standardised testing methods^14^. However, more efficient dark adaptometers have been developed and are now commercially available. For example, the AdaptDx dark adaptometer (MacuLogix, Hummelstown, PA) has been used in a number of clinical and research studies^3,14–17^. AdaptDx assesses the rod-intercept time (RIT), an estimate of the time duration for the rods to recover to an established criterion sensitivity (i.e. 5 × 10^−3^ scot cd/m^2^ (3 logarithmic units of attenuation of the stimulus)) after focal bleaching^17^. For example, when a photoflash bleaching approximately 83% of visual pigment is applied to a location 5° in the inferior vertical meridian, DA has subsequently been categorised as ‘normal’ if the RIT falls within the range of ≤ 12.3 min, or ‘impaired’ if longer^17^. This reference limit was determined by normative testing on young and old participants by Jackson et al., dictated by the protocol utilised^17^. A shorter duration protocol may be employed if the bleach intensity is reduced to 76%, whereby an adaptation time exceeding 6.5 min is considered to be ‘abnormal’, providing a diagnostic sensitivity and specificity for AMD both exceeding 90%^14^.

Assessment of DA in AdaptDx relies on a precise measurement of RIT. At times, especially for advanced AMD cases but sometimes even for those with early and intermediate AMD (iAMD), recovery cannot be recorded within a maximum allowed time for the test (usually 20–30 min)^3,18,19^. These are recorded either as censored data-points or as an estimated recovery time based on the trend observed from the data recorded within the maximum recording time. Therefore, RIT data represents a challenge for statistical analysis. Many authors use standard statistical approaches to analyse groups of RIT values such as a t-test^2, 5,15,16,20,21^ or non-parametric equivalents^3,11,14,18,20^. However, the t-test may not be appropriate when capping distorts the distribution of data. Furthermore, the value of non-parametric tests is limited by their relative lack of power and inability to generate confidence intervals (CI). Resampling methods, such as bootstrap techniques, could provide p-values and CIs without the distributional assumptions of asymptotic parametric tests. Yet these methods do not address the issue of bias in the estimates arising from truncation/censoring in the data. Another approach is to consider the failure to recover within the test time as a categorical variable^11^, although this limits the applicability of the analysis to longitudinal studies, where it is desirable to monitor a change in the variable over time.

We propose a ‘time-to-event’ analysis, commonly referred to as survival analysis, to be applied to RIT data. Time-to-event analysis is widely used in medical literature^22^ and is a method for assessing the length of time until the occurrence of a defined end-point of interest. Here we use the approach to describe the cumulative proportion of people within each group reaching the rod intercept as a function of time after cessation of the bleach. We hypothesise this method offers better statistical power than standard techniques when applied to these types of data. Potential gains could translate into fewer study participants (reduced sample sizes) for trials and studies using measures of DA. We used a previously published dataset to illustrate the method and test the hypothesis^19^. In addition, we developed and published a web-based app to implement this technique; this can be freely used by researchers and clinicians wanting to compare groups of people for which RIT values have been measured (https://bethanyelorahiggins.shinyapps.io/Time-to-EventAnalysis/).

## Results

Of those who participated in the previous study, 14 people with variable stages of AMD and eight age-similar controls provided valid data for the 76% bleach, 12° eccentricity test condition and were used to determine optimal test conditions for acquiring RIT with the AdaptDx instrument. The study reported no significant difference in age (mean controls: 69 years ± 8 standard deviation (SD); mean iAMD: 71 years ± 8 SD, independent samples t-test, p = 0.73) between control participants and those with AMD^19^. Table 1 summarises the demographic characteristics of the included participants.

**Table 1:** Clinical characteristics of all participants.

Participant ID	logMAR test eye	AMD status test eye	AMD status fellow eye	RIT (minutes)
RR0013	0.16	1	1	7.5
JE0008	0.00	1	1	6.3
JC0032	0.16	1	1	5.2
GM0035	− 0.04	1	1	1.8
BW0037	0.00	1	1	5.0
MI0033	0.16	1	1	2.7
SF0034	0.10	1	1	2.0
FJ0038	0.16	1	1	5.8
KM0003	0.16	2	2	3.6
DH0005	0.44	3	3	2.6
MM0006	0.20	3	3	6.7
GE0010	0.00	3	3	6.8
PS0012	0.20	3	3	6.9
GD0014	− 0.04	3	3	6.0
VC0015	0.02	3	4	10.2
PN0009	0.06	3	4	5.9
JB0018	0.00	3	3	10.1
WP0032	0.40	3	3	14.4
JG0027	0.20	4	4	12.3
EC0011	0.44	4	4	8.7
AF0028	0.50	4	4	3.2
PF0031	0.12	4	4	2.3

AMD graded according to the Beckman initiative severity scale. In short, eyes were grouped as normal ageing [1], early AMD [2], intermediate AMD [3], and late AMD [4] (geographical atrophy and/or neovascular lesions)^33^.

**Figure 1:**
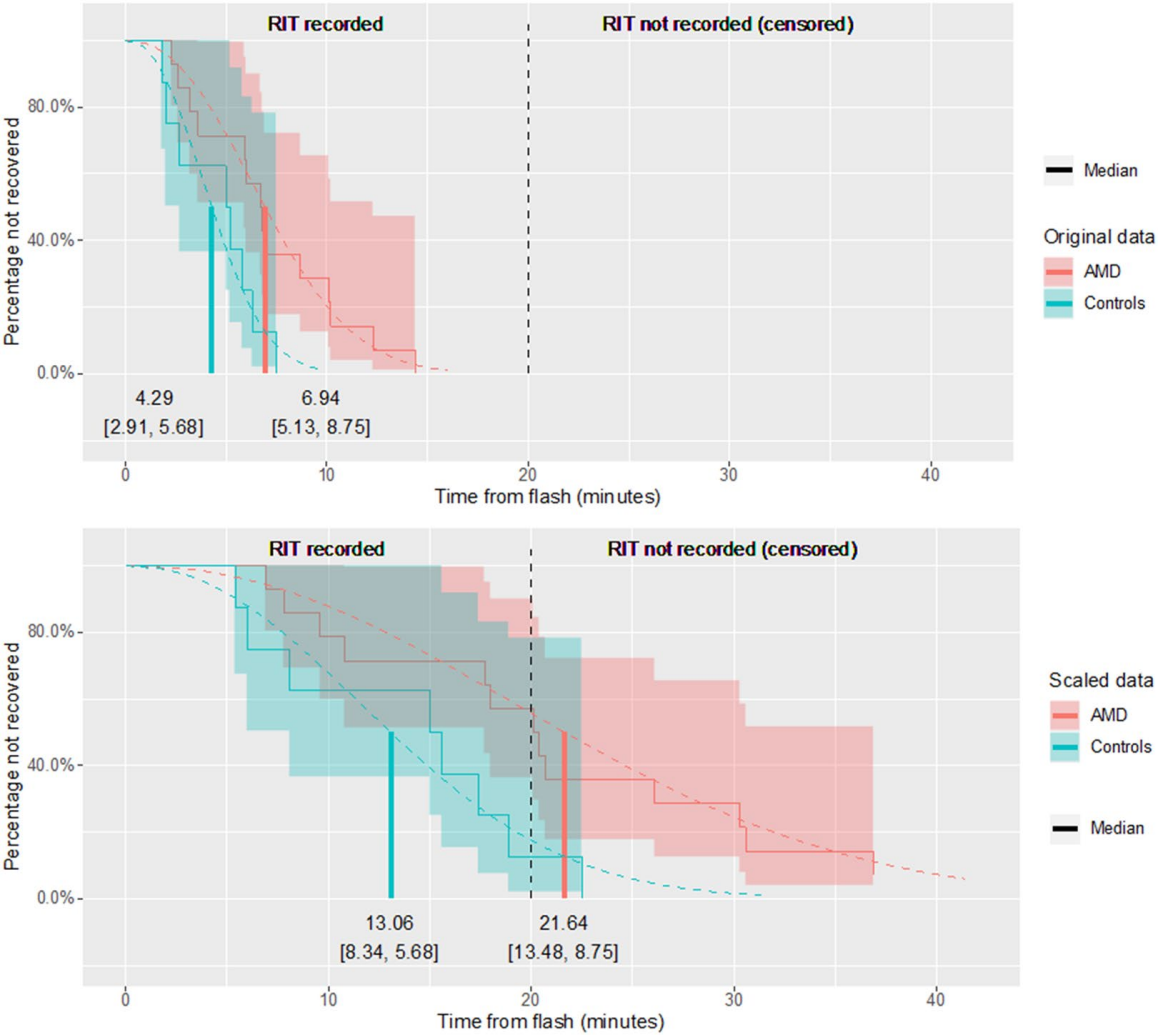
Empirical curves for the original (top) and the transformed (bottom) data, scaled to illustrate RITs that surpass a cut off time. The vertical dashed line acts as a marker, representing this capping limit of 20-min. Note that the actual underlying distribution of RIT values is depicted in the figure, so no censoring is shown here. However, the survival model is fitted considering all values beyond 20 min as censored. For the scaled data, both the fitted survival curves from the mode (dashed curves) and the median values (vertical solid lines) were calculated from capped data. However, the time-to-event model fits the data well even beyond the cut off time. For the scaled AMD data, the time-to-event model correctly predicts a median value beyond the capping limit. Figure generated using the ggplot2 package^32^.

Fitted curves are shown in Fig. 1. The central estimates for the three methods are reported in Table 2. A statistically significant difference (at p < 0.05) between groups was only detected with the time-to-event model (Table 2) in the original data. The p-values for the original and the scaled uncapped RITs were identical, the second being simply the same data scaled by a constant. With capped data, both the Generalised Linear Model (GLM) and the linear model yielded very biased estimates, especially in the AMD group (larger number of capped values). In contrast, the results of the time-to-event model were much closer to the values obtained without capping. With capped data, the CIs were larger for the time-to-event model but smaller for both the GLM and the linear model.

**Table 2:** Central estimates [95% confidence intervals (CIs)] of RIT values (in minutes) with the three methods considered (first and second columns).

	Estimate [95% CIs]		Effect [95% CIs]	p-value
	AMD	Controls		
**Original data**				
Survival model	6.94 [5.13, 8.75]	4.29 [2.91, 5.68]	1.62 [1.1, 2.37]	**0.014**
GLM	7.12 [5.26, 8.98]	4.54 [5.26, 8.98]	1.57 [1.02, 2.42]	0.055
Linear model	7.12 [5.44, 8.81]	4.54 [5.44, 8.81]	2.58 [− 0.21, 5.38]	0.085
**Scaled data, uncapped**				
Survival model	20.81 [15.38, 26.25]	12.88 [8.72, 17.03]	1.62 [1.1, 2.37]	**0.014**
GLM	21.36 [15.78, 26.95]	13.61 [15.78, 26.95]	1.57 [1.02, 2.42]	0.055
Linear model	21.36 [16.31, 26.42]	13.61 [16.31, 26.42]	7.75 [− 0.62, 16.13]	0.085
**Scaled data, capped**				
Survival model	21.64 [13.48, 29.79]	13.06 [8.34, 17.79]	1.66 [0.97, 2.84]	0.066
GLM	16.49 [13.33, 19.64]	13.3 [13.33, 19.64]	1.24 [0.9, 1.7]	0.199
Linear model	16.49 [13.63, 19.34]	13.3 [13.63, 19.34]	3.19 [− 1.55, 7.92]	0.202

For the linear model (t-test) and the GLM, the mean is reported. For the survival model the estimate for the median is reported. The third column reports the effect [95% CIs] measured by the three methods, which is the basis for the calculation of the p-value. Significant p-values at an alpha level of 0.05 are shown in bold. The effect is the ratio between the mean RITs of AMD and controls for the survival model and the GLM, and the difference between the two groups for the linear model. Notice how the estimate from the survival model is much less affected by capped values.

**Figure 2:**
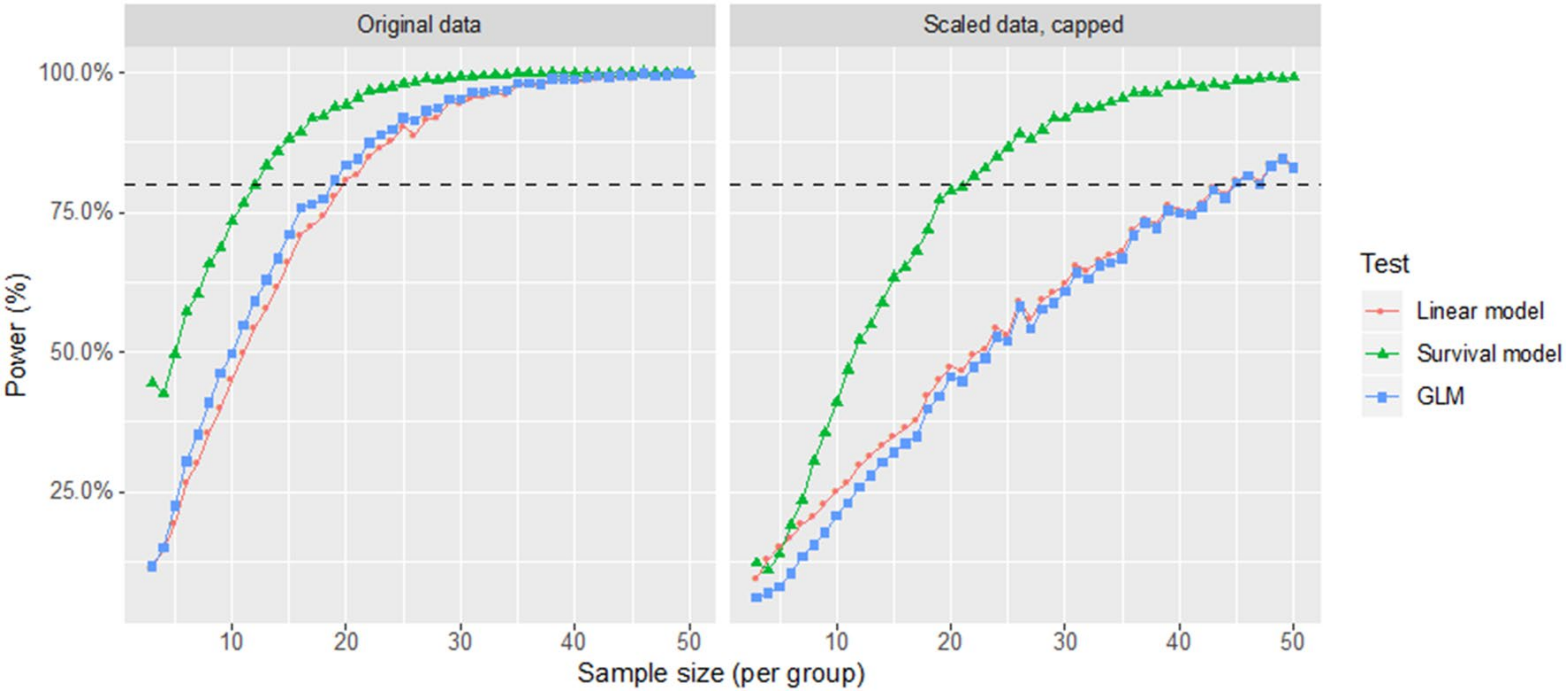
Power curves as a function of sample size for the three methods considered with and without capping for the scaled data. The power curves for the original data (not scaled) are identical to the scaled uncapped data and are therefore not reported (10,000 bootstrap simulations for each step in the sample size). Figure generated using the ggplot2 package^32^.

When we investigated the power of the three methods via bootstrap, the time-to-event model was superior. This is demonstrated by the power curves as a function of sample size in Fig. 2. The number of subjects needed per group to detect a significant difference (α = 0.05) at 80% power are reported in Table 3. When censored observations were introduced, the power of all methods was decreased, but the time-to-event model still performed better than the linear model and GLM, and this is noteworthy. The estimated effect was much less affected by capping with the time-to-event model compared to the other two methods; this offers a considerable practical advantage in studies where participants’ RIT could exceed the maximum time set in a protocol.

**Table 3:** Sample size (to whole number) of each group required to reach 80% power at α = 0.05 with the three methods considered.

	Uncapped data	Capped data
	Sample size at 80% power (per group)	Sample size at 80% power (per group)
Survival model	12	21
GLM	18	44
Linear model	20	45

A supplementary analysis was conducted to further demonstrate the applicability of our proposed methodology in a second, censored dataset^19^. Sixteen people from the same cohort used for the main analysis with various stages of AMD and eight age-similar controls provided valid data for the 76% bleach, 5° eccentricity test condition. This dataset features censored data because three RITs were not accrued within the test time and were capped at 30-min. Demographic characteristics of the included participants in this second analysis can be found in Supplementary Table S1 online. In this supplemental dataset, it is clear that the survival analysis offers a smaller p-value when testing the difference between the two groups, compared to both the GLM and linear model. This result is in line with the improved statistical power shown with our bootstrap experiment. Furthermore, the CIs for the central estimates are wider, exemplifying that censored data only provide limited information. However, as for our main calculations, accounting for censored data provides larger differences because it reduces the downward bias induced by capped data. See Supplemental Table S2 and Fig. S1 online for details.

## Discussion

We have shown how time-to-event analysis can be applied to the data yielded from psychophysical measurements of DA. Compared to alternative statistical methods, the proposed time-to-event model achieved higher statistical power in discriminating between people with AMD and healthy controls. Our method is statistically correct, by which we mean it can accommodate for both skewed data and censored data points. Time-to-event analysis offers the advantage of significantly reducing sample sizes when planning studies where this functional test is an outcome measure. The latter is important because designing trials and studies more efficiently equates to newer treatments likely being examined more efficiently. Our method may also have application to longitudinal studies and trials such as evaluation of proof-of-concept or phase II clinical trials aimed at early intervention. Moreover, this model offers flexibility and allows for additional covariates to be added to the analysis (e.g. presence of pseudodrusen or age), making a wide range of RIT analysis possible. We have made the technique freely available via a simple App.

Both the GLM and the time-to-event model are able to account for the skewed distribution of the data; the former employs a Gamma distribution for the error, whereas the latter makes use of a Weibull distribution. However, time-to-event analysis can also accommodate censored observations. This feature is expected to prove useful for the assessment of DA impairment in people with AMD because examination time is usually capped at a maximum for practical reasons e.g. 20–30 min; a deficient RIT may exceed the maximum time of the test^16^. This issue has been addressed in different ways in previous studies, for instance simply using the capped value as if it was an actual measured RIT^16,17,19,21^. We adopted the same solution for our simulated capping when using the GLM and the linear model. Of course, the major drawback of artificial capping is that it will create a false peak at the capped value. This is similar to what is observed in sensitivities in visual field examination with standard automated perimetry, where thresholds below 0 dB cannot be tested, resulting in a zero inflated distribution^23^. With our results, we showed that such an approach can severely bias the central estimates (Table 2). For example, in the scaled capped data for people with AMD (the group with largest amount of capped values), the estimates for the mean are much smaller than the correct values obtained from uncapped data. Moreover, the CIs are narrower with capped data and do not include the correct value for the mean. In contrast, the estimate of the median from the time-to-event model is very close to the value calculated without capping. The CIs are also wider, correctly reflecting the fact that censored data only provide partial information. Finally, the time-to-event model can correctly predict a median value beyond the capping limit (20-min). This would not be possible with a raw calculation of the median.

One alternative solution to deal with capped data is to use values estimated from the DA recovery curve. The AdaptDx is able to fit a decay model to the acquired values and extract a RIT value by projecting the estimated curve forward in time. This is allows for missing data points and has been employed by some researchers^3^. However, this is subject to the assumptions of the fitted curve and to measurement variability in the acquired data; it is unable to fit the decay model where limited recovery has taken place within the duration of the test, and thus capped data points still remain.

Our technique should have wide application in the context of studies measuring DA. For example, functional deficit in DA has been shown to become apparent before other clinical measures of visual function are affected^16,24^. Moreover, evidence suggests that delayed DA may manifest before the appearance of structural features of AMD such as drusen and focal pigmentary changes^1–9^, indicating that DA is a pertinent clinical measure. Indeed, a series of studies, of varying quality, have shown a measure of DA to be a diagnostic indicator of AMD^9,13,17,24^. The rate of DA has been shown to increase with increasing severity of AMD^3,11,14,18^.

The unusual statistical properties and subsequently skewed distribution of RIT values has been largely overlooked in previous reports^5,20^. This can have important negative effects on the power of statistical tests, as illustrated by our power analysis (Fig. 2). In many cases, researchers resorted to non-parametric tests, acting on the ranks of the data, because they do not make assumptions on the distribution of the data. However, classical non-parametric tests are less powerful than their parametric alternatives and they do not provide CIs on the estimates.

There are some limitations to what we have proposed. For the purposes of this study, we did not attempt to distinguish between stages of AMD. However, the use a disease vs. non disease dataset was sufficient to demonstrate the reduction in sample size associated with the statistical techniques used, and the methods would be equally applicable to studies designed to discriminate between different disease severities. Our method is primarily meant to compare RIT values among groups of people involved in a study or clinical trial. In fact, it is focused on the estimation of group effects as global changes in time scale of recovery and would provide little information on individual subjects. Future work could focus on the application of our methodology to larger datasets and longitudinal data; we hope our App for using this technique will help facilitate this.

Another perceived limitation of our study is our use of a dataset that does not feature RITs > 15-min and scaling the dataset to reflect censored data. However, this allowed us to demonstrate the strength of the time-to-event method by showing how the estimates obtained with capped data would compare to those obtained from fully measured RITs. Such a comparison would have been impossible had censoring been present in the original data, because the true underlying distribution of RITs would have been unknown. However, we have also included a supplemental analysis on a second dataset with actually censored RIT values to further highlight the real-life applicability of this methodology.

The RIT measurement itself has limitations as it is not only dependent on DA kinetics but also on parameters such as pupil size and the number of photoreceptors (known to vertically scale sensitivity). Analysis of other metrics of DA measurements such as slope of the S2 component may be more demonstrable of DA kinetics^12,13,25^. Furthermore, the intent of this report was only to assess the RIT as produced by the analysis responses from the machine. We have not reanalysed the responses themselves in order to offer a different strategy for estimating the RIT. Larger datasets would also provide the opportunity to test other distributions for our time-to-event analysis. In fact, despite being widely used in parametric time-to-event analyses for its flexibility, the Weibull distribution might not necessarily be the best choice for this type of data. Finally, as explained in the methods section, these models do not describe the data in exactly the same way: both the GLM and the time-to-event model perform the comparisons in the logarithmic scale. This implies that, opposite to the linear model, they model the changes as proportions rather than linear differences. This is a common choice in many fields where strictly positive values are expected (such as with RIT values) since this data usually exhibit a heteroscedastic behaviour whereby the variance increases with the predicted mean. Log-scale models account for this behaviour^26^. Moreover, the logarithmic scale reduces the influence of large values which would otherwise greatly affect the mean calculated in the linear scale.

One final important aspect is that different conventions to calculate p-values are used for survival analyses (Wald test) and linear models/GLMs (t-test). We address this issue in a supplemental analysis, where we show that the improvement in power obtained with the time-to-event analysis is unchanged when the p-values are calculated using the Wald test for all the models (See Supplementary Fig. S2). In summary, the use of a time-to-event analysis is a more powerful statistical measure compared to other statistical approaches, for the assessment of RITs of people with AMD. We propose that time-to-event curves are a useful tool to visualise RIT in groups of people. We make full use of this in our freely available app, providing a user-friendly interface for clinical scientists to visualise and analyse RIT data more efficiently.

## Methods

### Participants

We retrospectively analysed data collected for a previous study by Binns et al.^19^. Institutional research ethical approval was approved by School of Health Sciences, City, University of London. All procedures adhered to the tenets of the Declaration of Helsinki and were carried out in accordance with relevant guidelines and regulations. All the data were anonymised for this study and informed consent was obtained from all subjects. Details on recruitment and inclusion/exclusion criteria can be found in the original paper^19^. In brief, age-similar visually healthy controls and people with early AMD, iAMD and non-central geographic atrophy were recruited. Inclusion criteria consisted of best corrected visual acuity of logMAR 0.7 or better in study eye, > 55 years of age, adequately clear ocular media and acceptable pupillary dilation and fixation to allow for quality fundus photography. Exclusion criteria included significant disease, other retinal pathology in the study eye, or a history of medication known to disturb visual function^19^. For the scope of our analysis, we did not distinguish between different stages of AMD.

### Dark adaptation procedure

For the purposes of our work, we used the values obtained with the optimal testing conditions as determined by Binns et al. (76% bleach at 12° eccentricity)^19^. Full details of the DA procedure have been published previously^19^. Briefly, prior to assessment the participant was dark adapted for 30-min in a darkened room. An appropriate spherical lens was used (+ 3.00 DS plus spherical distance prescription) and a patch placed over the non-tested eye. The participant then viewed a fixation stimulus from a chin rest. Alignment was monitored using an infra-red camera and adjusted by the examiner. Pupil diameter was measured before the administration of the bleaching, 505 nm bleaching flash (4° diameter, centred 12° in the inferior visual field, 0.8 ms duration, 1.8 × 104 scot cd/s.m2, bleaching an estimated 76% of rod visual pigment^27^). The test stimulus was subsequently shown at the same location as the bleach. Fifteen seconds after the photoflash, the threshold was measured for a 505-nm, 2° diameter target starting. The participant was asked to keep looking at the fixation light and to press a response button when a flashing target became visible. A modified staircase procedure was utilised to estimate the threshold until the RIT was attained or the cut-off time was reached (30-min). A 15-s break was given after each threshold. If the RIT was not reached within the test, it was set at the maximum test duration (30-min). As in previous studies utilising the AdaptDx^14^, if fixation errors exceeded 30% of threshold points, the test was deemed unreliable^19^.

To further demonstrate the applicability of our methodology, the same analysis was conducted on a second, supplementary dataset obtained from Binns et al.^19^ (76% bleach at 5° eccentricity). The DA protocol used was identical except the 505 nm bleaching flash was centred at 5° in the inferior visual field.

### Time-to-event analysis

**Figure 3:**
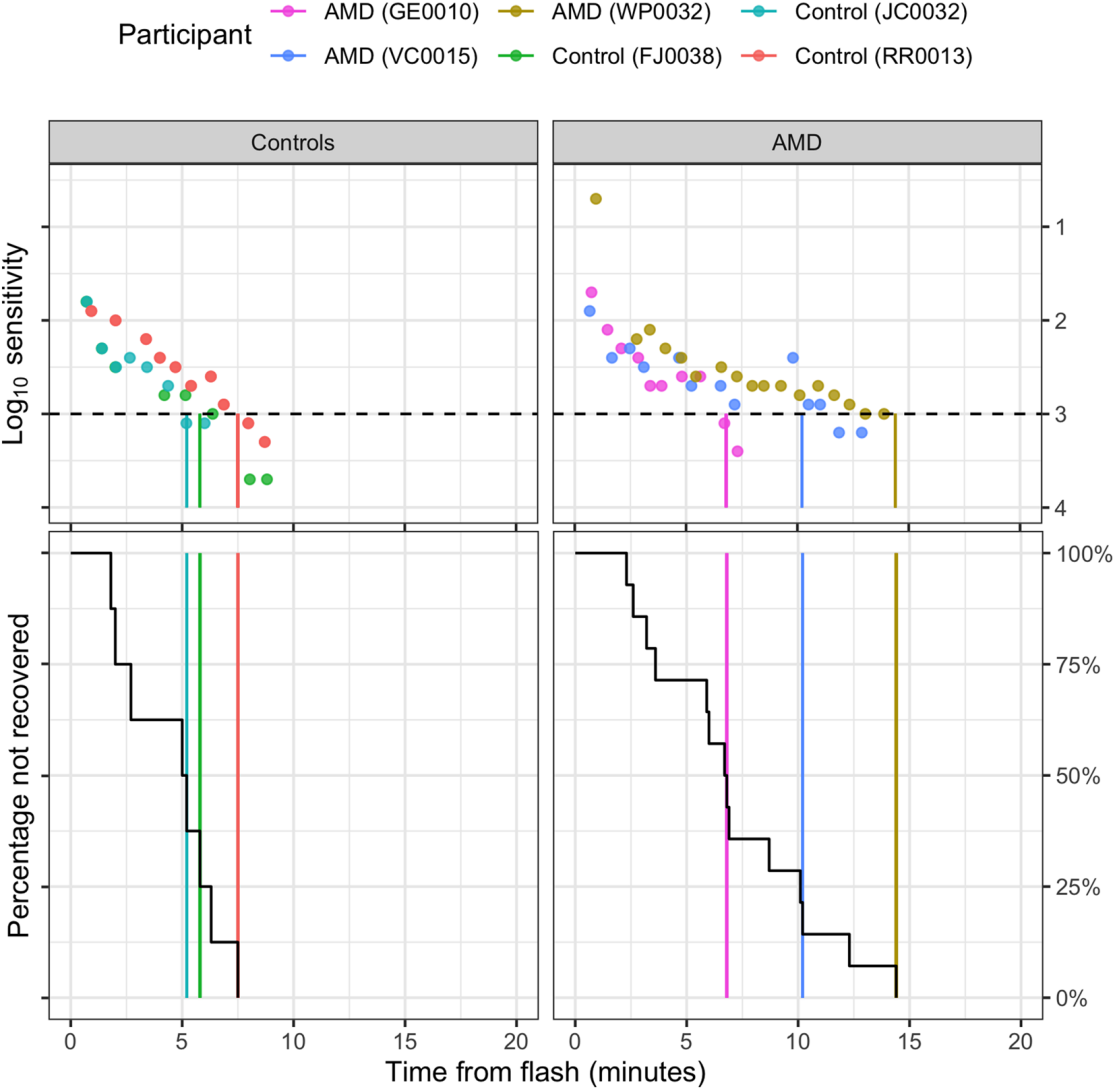
The change in Log_10_ sensitivity for three control subjects and three patients with AMD is plotted in the top panel (filled dots) with the corresponding Rod Intercept Time values (vertical strokes) provided by the device. The horizontal dashed line represents the 3 log-step change in sensitivity used by the device to define the event (recovery from bleaching). The RIT time for each recovery “event” is used to build the survival curves (bottom panels). In this case, the vertical coloured lines also identify the same RIT values recorded for the curves plotted in the top panels. Notice how each vertical line corresponds to a downward step change in the survival curve (in black). The same process is applied for all RIT values in the dataset to calculate the other step changes that make up the rest of the survival curve. A marker demonstrating the cut off time has been added. Figure generated using the ggplot2 package^32^.

We used a parametric time-to-event model widely used in medical literature to describe the time taken for an event such as tumour recurrence or time to death after a treatment^22^. Here we use the approach to describe the cumulative proportion of people within each group reaching the rod intercept as a function of time after cessation of the bleach. RIT was not treated as the event itself, but rather the time taken for the participant to recover sensitivity to a stimulus intensity of 5 × 10^−3^ scot cd/m^2^ (a decrease in threshold of 3 logarithmic units). In this respect, the event recovery can be used in a time-to-event analysis, since the RIT is for all intents and purposes the time passed until such an event is observed. In other words, we model RIT values within each group as the cumulative occurrence of recovery over time; a cumulative distribution function *F(t)*. RIT values can be plotted as survival curves (see Fig. 3) using a Kaplan–Meier estimator^28^. These curves report the time from bleaching on the horizontal axis and the percentage of recovered subjects on the vertical axis. This is a step graph and changes occur at each observed RIT (downward step). Censored data can be represented with a marker, as shown in Fig. 1 and Supplemental Fig. S1. An example of how the survival curve can be plotted from RIT values is reported in Fig. 3.

Its inverse is the time-to-event function *S(t)* = *1 – F(t)*. The function F(t) can be modelled as the cumulative distribution function of a variety of distributions. One of the most common is the Weibull distribution, which is then assumed to be the distribution of the observed times-to-event. This is called Accelerated Time Failure model (Eq. 1).

1 $$\log \left( T \right) = \beta_{0} + \beta_{1} *Group + \sigma W$$

Here $T$ represents the time to recovery, Group denotes the assignment of the subject *i* (for example AMD or Control), $\sigma$ denotes a scale factor for the errors and $W$ is the assumed distribution that has F(t) as cumulative distribution function (Weibull in this case).

Additional predictors and interactions can be added to the right hand side of the function if needed as in a multivariable linear regression (a treatment arm, for example). In our scenario, the expectation is the AMD group will have a proportional increase in “time-to-event time” since more time is needed for the event (recovery from bleaching) to happen, i.e. longer RIT values. One advantage of parametric time-to-event models (as the one proposed) over a semi-parametric Proportional Hazard model (Cox model) is that the baseline time-to-event function is explicitly modelled, thus allowing estimates and inference on time-to-event times. Moreover, delayed/accelerated recovery in one group with respect to the baseline level (for example, AMD with respect to Controls) can be simply calculated as ${\mathrm{exp}(\beta }_{1})$, where exp() denotes the exponential function. So, for example, a value of ${\beta }_{1}=0.7$ indicates a two-fold increase in recovery time.

Handling censored data is a key feature of time-to-event analysis^29^. In our application, right-censoring happens when the RIT is longer than the maximum time allowed for the test; this can be denoted using a binary variable, commonly assuming a value of 1 when the RIT has been recorded and a value of 0 otherwise. In our dataset the maximum recording time was set at a lengthy 30 min^19^; no subject exceeded this limit. In other studies, especially in a clinical use scenario, this limit is likely lower, e.g. 20-min (Fig. 1). Therefore, in order to demonstrate how the time-to-event analysis can be used with censored data, we transformed the data with a multiplicative constant (c = 3) and subsequently capped the data at 20-min. This allowed us to explore how the estimates from different modelling approaches change between the capped and the full series. In this specific case the censoring is non-random, as it is set by a predetermined stopping time. The strength of time-to-event analysis is that, using censoring, it is still possible to extract information from unrecorded RIT values as time-to-event analysis can account for the fact that, at termination time (20-min), a percentage of subjects have not recovered from bleaching. By contrast, any other method would require to either exclude participants with unrecorded RIT values or to impute the values for the missing data.

### Other parametric methods

The time-to-event model was compared with two parametric models, the t-test and a GLM. Both make strong assumptions concerning data structure, such as the independence of each data point and the correct scale of the data. However, the GLM can better accommodate for skewed error distributions. Of course, the former can be interpreted as simple linear model, where the predictor is a binary factor with only two classes (Group). It can be formulated as Eq. (2).

2 $$RIT_{i} = \beta_{0} + \beta_{1} *Group_{i} + \epsilon_{i}$$

In this case, the response variable is the RIT for the subject *i*, the parameter ${\beta }_{0}$ (Intercept) represents the mean RIT for the baseline Group (Controls in this case), the parameter ${\beta }_{1}$ represents the estimated difference between the two groups, and ${\epsilon }_{i}$ is the error, assumed to be Gaussian.

This linear model formulation can be extended to GLM, which uses a link function for the mean of the response^26^ (in this specific case, the natural logarithm). This effectively allows the model to have a Gamma distribution (instead of Gaussian) for the error, accounting for the skewed distribution of the data. Note that this is different from a log-transformation of the data: the link function is applied to the *mean* of the response and is therefore invertible, i.e. the inverse log of the mean response from the GLM produces the corresponding estimate of the RIT in the linear scale. On the contrary, with a log-transformation of the data, the model will estimate the mean of the log-response, which cannot be converted back to the mean of the original response. For both these models, censored data are replaced by the maximum recordable value (20 min or 30 min).

### Power calculation

Power calculations were used to compare the efficiency of the different statistical approaches. To avoid distributional assumptions on the real data as much as possible, we used a bootstrap procedure to estimate the power of the three methods at different sample sizes. We used random sampling, with replacement, with N subjects from the controls and the same number from the AMD group. Due to replacement, the same subject could be extracted multiple times and arbitrarily large samples could be produced. At each extraction, the three methods were applied and the p-value on the null hypothesis of no difference between RIT in people with AMD and controls from each method was recorded. The sampling was repeated 10,000 times at different sample sizes (N from 3 to 50 per Group). The power for each value was calculated as the proportion of extractions where the p-value was below 0.05. As a clarification, the bootstrap was not used to calculate the p-value, which was instead derived from each parametric test, but just to generate the random samples on which the tests were performed. We have used a similar approach in previously published work^30^ to perform a post-hoc power calculation.

For our main analysis, when computing the p-values, we adopted the statistical convention for each model: t-test for the parameter derived from the linear model and GLM and the Wald test for the parameters derived from the survival analysis model^31^. However, to prove that the differences in power between the three methodologies were not due a different calculation of the p-values, we performed an additional power analysis using the Wald test for all the models.

### Web application

We designed a purpose written, interactive application to demonstrate the time-to-event analysis technique for RIT data. The application uses Rstudio's Shiny framework and is available in the public domain. It allows a user to upload their own RIT data in .csv format to use the statistical test and produce a time-to-event plot to illustrate the data. The application has the option to use the data illustrated in this paper. (https://bethanyelorahiggins.shinyapps.io/Time-to-EventAnalysis/).

All analyses were performed in R 3.5.2 (http://www.r-project.org/) under R Studio, version 1.1.463 (RStudio, Boston, MA, USA). For time-to-event analysis the parametric time-to-event regression provided in the Survival package for R was implemented^28^. Figures were generated using the ggplot2 package^32^.

## Supplementary Information

Supplementary Information.

## Data Availability

The application and hence analysis used in this paper can be accessed through https://bethanyelorahiggins.shinyapps.io/Time-to-EventAnalysis/. All data used in this paper was sourced from Binns et al.^19^.

## Code availability

The application and hence analysis used in this paper can be accessed through: (https://bethanyelorahiggins.shinyapps.io/Time-to-EventAnalysis/), the code details will be made available on direct request to the author.
