# Supplementary material for: Optimising assessment of dark adaptation data using time to event analysis
Source: Sci Rep. 2021 Apr 15;11:8323. doi: 10.1038/s41598-021-86193-3 (PMC8050245; doi:10.1038/s41598-021-86193-3)
Supplement: Supplementary file 1 — Supplementary Information. [file 41598_2021_86193_MOESM1_ESM.docx]

**Optimising Assessment of Dark Adaptation Data Using Time to Event Analysis**

**Bethany E Higgins^1^, Giovanni Montesano^1, 2^, Alison M Binns^1^, David P Crabb^1*^**

1. Optometry and Visual Science, School of Health Sciences, City, University of London Northampton Square, London, United Kingdom, EC1V 0HB
2. Moorfield’s Eye Hospital NIHR Biomedical Research Centre, Moorfields Eye Hospital NHS Foundation Trust and UCL Institute of Ophthalmology, London, UK

*Corresponding author: Professor David P Crabb; Email: david.crabb.1@city.ac.uk; Phone: +44 207 040 0191

**Supplementary Materials**

| **Participant ID** | **logMAR test eye** | **AMD status test eye** | **AMD status fellow eye** | **RIT (minutes)** |
| --- | --- | --- | --- | --- |
| RR0013 | 0.16 | 1 | 1 | 6.5 |
| ET0007 | 0.34 | 1 | 1 | 5.1 |
| JE0008 | 0.00 | 1 | 1 | 4.1 |
| JC0032 | 0.16 | 1 | 1 | 3 |
| GM0035 | -0.04 | 1 | 1 | 12.8 |
| BW0037 | 0.00 | 1 | 1 | 3.1 |
| MI0033 | 0.16 | 1 | 1 | 21.5 |
| FJ0038 | 0.16 | 1 | 1 | 7.5 |
| AG0002 | 0.20 | 2 | 2 | 12.3 |
| KM0003 | 0.16 | 2 | 2 | 14.5 |
| DH0005 | 0.44 | 3 | 3 | 10.8 |
| MM0006 | 0.20 | 3 | 3 | 7.6 |
| GE0010 | 0.00 | 3 | 3 | 12 |
| PS0012 | 0.20 | 3 | 3 | 30* |
| GD0014 | -0.04 | 3 | 3 | 29 |
| VC0015 | 0.02 | 3 | 4 | 30* |
| BB0016 | 0.42 | 3 | 4 | 30* |
| PN0009 | 0.06 | 3 | 4 | 7.3 |
| JB0018 | 0.00 | 3 | 3 | 12.3 |
| WP0032 | 0.40 | 3 | 3 | 30* |
| JG0027 | 0.20 | 4 | 4 | 4.6 |
| EC0011 | 0.44 | 4 | 4 | 10.6 |
| AF0028 | 0.50 | 4 | 4 | 14 |
| PF0031 | 0.12 | 4 | 4 | 3.8 |

* RIT was not reached within the maximum test duration. These data were censored and a value of 30-minutes was allocated.

**Supplementary Table S1.** Clinical characteristics of participants included in supplementary analysis. These values were obtained by Binns et al (2018) using a DA procedure consisting of 76% bleach at 5° eccentricity, detailed in manuscript ^1^. AMD was graded according to the Beckman initiative severity scale. In short, eyes were grouped as normal ageing [1] , early AMD [2], intermediate AMD [3], and late AMD [4] (geographical atrophy and/or neovascular lesions) ^2^.

|  | | **Estimate [95% CIs]** | | |
| --- | --- | --- | --- | --- |
|  | | **AMD** | **Controls** | **p-value** |
| **Original data** | **Survival model** | 16.37 [9.62, 23.12] | 6.74 [3.17, 10.32] | 0.007 |
|  | **GLM** | 16.18 [10.8, 21.55] | 7.95 [10.8, 21.55] | 0.024 |
|  | **Linear model** | 16.18 [11.79, 20.56] | 7.95 [11.79, 20.56] | 0.045 |

**Supplementary Table S2.** Central estimates of RIT values (in minutes) with the three methods considered for the supplementary analysis. For the linear model (t-test) and the GLM, the mean is reported. For the survival model the estimate for the median is reported. In this supplemental dataset censored at 30-minutes, it is clear that the survival analysis offers a more statistically significant difference (at p<0.05) between groups compared to the GLM and linear model.

**
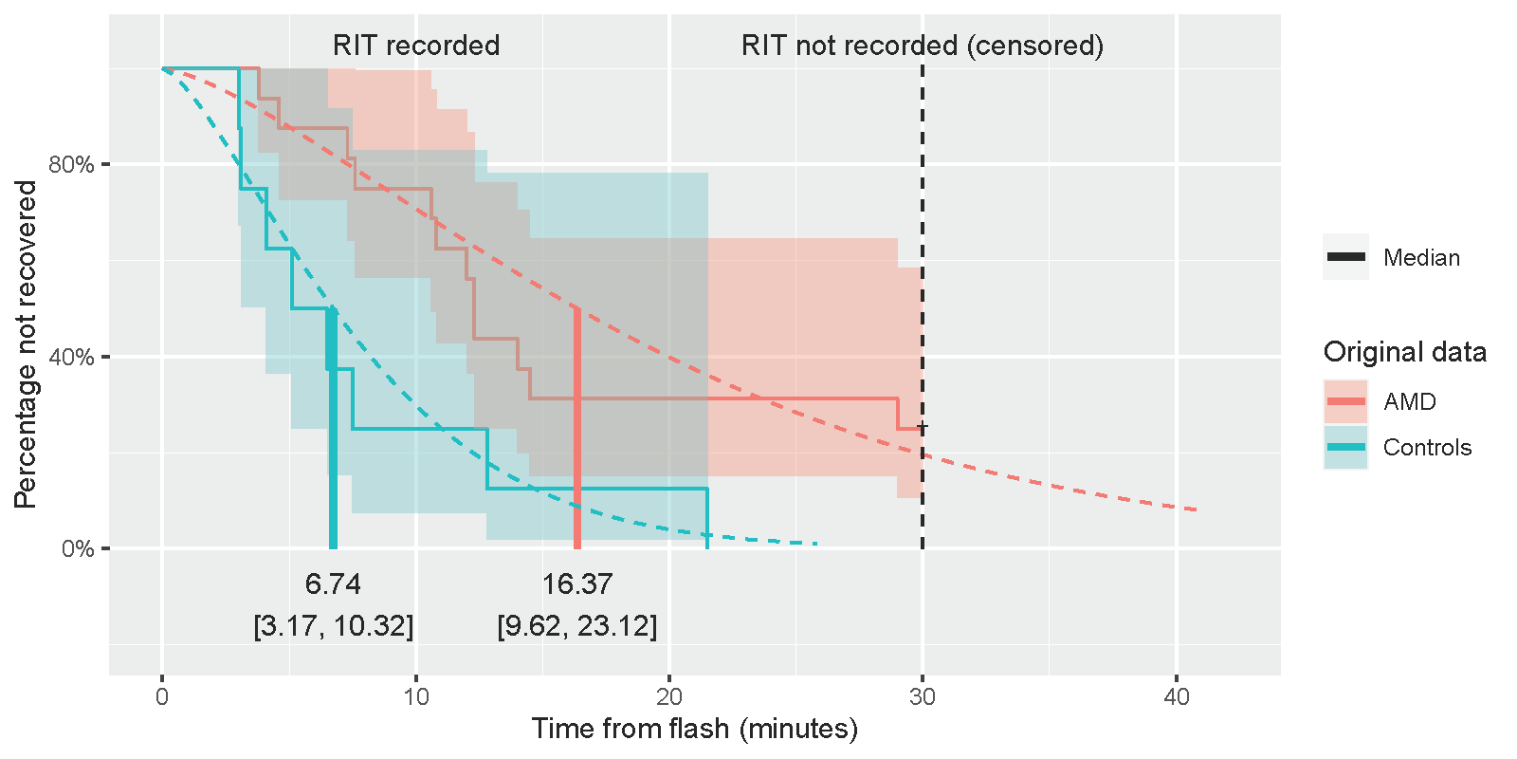
**

**Supplementary Figure S1.** Survival curves of supplementary dataset RITs. The vertical dashed line acts as a marker, representing this capping limit of 30-minutes. Note that the empirical survival curve for AMD eyes does not reach 0, showing that the values beyond 30 minutes are censored. The time-to-event model predicts a median value beyond the capping limit (shown by the extended red dashed line). Figure generated using the ggplot2 package ^3^.

**Supplementary Figure S2.** Power curves calculated using the p-values from the Wald test for all three models (N = 10000 bootstrap samples per sample size step). Figure generated using the ggplot2 package ^3^.

**Supplementary References**

1. Binns, A. M., Taylor, D. J., Edwards, L. A. & Crabb, D. P. Determining optimal test parameters for assessing dark adaptation in people with intermediate age-related macular degeneration. Investigative Ophthalmology and Visual Science 59, AMD114–AMD121 (2018).
2. Ferris, F. L. et al. Clinical classification of age-related macular degeneration. Ophthalmology 120, 844–851 (2013).
3. Wickham, H. *ggplot2: Elegant Graphics for Data Analysis. Springer-Verlag New York*. Available at <https://ggplot2.tidyverse.org> (2016).
